# Separation of Channels Subserving Approach and Avoidance/Escape at the Level of the Basal Ganglia and Related Brainstem Structures

**DOI:** 10.2174/1570159X21666230818154903

**Published:** 2023-08-24

**Authors:** Véronique Coizet, Racha Al Tannir, Arnaud Pautrat, Paul G. Overton

**Affiliations:** 1 Grenoble Institute of Neuroscience, University Grenoble Alpes, Bâtiment E.J. Safra - Chemin Fortuné Ferrini - 38700 La Tronche France;; 2 Department of Psychology, University of Sheffield, Sheffield, United Kingdom

**Keywords:** Basal ganglia, cerebral cortex, brainstem, neurotransmitter, striatum, approach and avoidance

## Abstract

The basal ganglia have the key function of directing our behavior in the context of events from our environment and/or our internal state. This function relies on afferents targeting the main input structures of the basal ganglia, entering bids for action selection at the level of the striatum or signals for behavioral interruption at the level of the subthalamic nucleus, with behavioral reselection facilitated by dopamine signaling. Numerous experiments have studied action selection in relation to inputs from the cerebral cortex. However, less is known about the anatomical and functional link between the basal ganglia and the brainstem. In this review, we describe how brainstem structures also project to the main input structures of the basal ganglia, namely the striatum, the subthalamic nucleus and midbrain dopaminergic neurons, in the context of approach and avoidance (including escape from threat), two fundamental, mutually exclusive behavioral choices in an animal’s repertoire in which the brainstem is strongly involved. We focus on three particularly well-described loci involved in approach and avoidance, namely the superior colliculus, the parabrachial nucleus and the periaqueductal grey nucleus. We consider what is known about how these structures are related to the basal ganglia, focusing on their projections toward the striatum, dopaminergic neurons and subthalamic nucleus, and explore the functional consequences of those interactions.

## 1 INTRODUCTION

The basal ganglia have been suggested to have an important role in selecting actions for a given context, with bids for selection coming *via* inputs from many structures [1]. Numerous experiments have studied action selection in relation to inputs from the cerebral cortex. However, from an evolutionary point of view, the basal ganglia are ancient structures. They appear in the nervous systems of all classes of jawed vertebrates [2, 3] and possibly in all vertebrates [4]. Most importantly, their cell types, intrinsic neurochemistry and patterns of connectivity have been highly conserved throughout vertebrate evolution. This means that the basal ganglia, in more or less their present form, were in place prior to the evolution of the cerebral cortex. We might, therefore, expect subcortical sensorimotor structures to be connected to the basal ganglia like that of the later evolving cortical sensorimotor regions and share a functional role in the basal ganglia action selection alongside the cortex [5]. Despite this, less is known about the anatomical and functional link between the basal ganglia and the brainstem. The brainstem is classically described as a major target of the basal ganglia *via* their output nuclei [6]. In this review, we describe how brainstem structures also project to the main input structures of the basal ganglia, namely the striatum, the subthalamic nucleus and midbrain dopaminergic neurons, in the context of approach and avoidance (including escape from threat), two fundamental, mutually exclusive behavioral choices in an animal’s repertoire in which the brainstem is strongly involved [5]. After a brief description of the basal ganglia and its role in action selection, we will focus on three particularly well-described loci involved in approach and avoidance, namely the superior colliculus, the parabrachial nucleus and the periaqueductal grey nucleus. We consider what is known about how these structures are related to the basal ganglia, focusing on their projections toward the striatum, dopaminergic neurons and subthalamic nucleus, and explore the functional consequences of those interactions.

## 2 THE BASAL GANGLIA

### 2.1 Anatomy of the Basal Ganglia

The basal ganglia are a group of interconnected subcortical nuclei that represent one of the brain’s fundamental processing units. The basal ganglia are a loosely grouped collection of sub cortical nuclei derived from the telencephalon and midbrain, located deep within each cerebral hemisphere. The principal components of the basal ganglia are the striatum (caudate nucleus, the putamen and ventral striatum), the subthalamic nucleus, globus pallidus and substantia nigra (pars reticulata and pars compacta).

The main “input” components of the basal ganglia are the striatum and the subthalamic nucleus [7]. Afferent connections to both structures originate from virtually the entire cerebral cortex (including motor, sensory, association and limbic areas) [8], from the mid-line and intralaminar nuclei of the thalamus and the limbic system (principally the amygdala and hippocampus). These connections are excitatory, intermittently active, and use glutamate as a neurotransmitter [9-11].

The main “output nuclei” of the basal ganglia are the substantia nigra (pars reticulata) and the entopeduncular nucleus (internal segment of the globus pallidus in primates). These structures provide extensively branched efferents to the thalamus (which in turn projects back to the cerebral cortex) and pre-motor areas of the brainstem, including the superior colliculus, the periaqueductal grey nucleus, the parabrachial nucleus, the pedunculopontine/cuneiform area, and widespread regions of the mesencephalic/medullary reticular formation [6, 12-14]. Most output projections are tonically active and inhibitory and use GABA as a neurotransmitter.

The “intrinsic connections” of the basal ganglia are organized so that phasic input entering the system can either decrease or increase the tonic inhibitory effect that the GABAergic output nuclei have on their target structures [15]. Selective disinhibition of areas receiving inputs from basal ganglia output structures is promoted by direct inhibitory connections between striatal cells and the output nuclei, suppressing tonic output firing (“direct pathway”) and thereby releases output targets in the thalamus and brainstem from being tonically inhibited. In contrast, external afferents to the subthalamic nucleus (the “hyper-direct pathway”), which projects directly *via* excitatory connections to the output nuclei, can increase the level of tonic inhibitory control over the thalamus and brainstem [16]. Tonic inhibitory control can also be increased when subthalamic nucleus activity is increased following striatal inhibition of inputs from the external globus pallidus (“indirect pathway”). Further intrinsic processing is provided by dopaminergic projections from the ventral midbrain (substantia nigra pars compacta and ventral tegmental area) to the striatum. Mesencephalic dopaminergic neurons, in turn, receive a direct inhibitory projection from the striatum and excitatory input from the subthalamic nucleus.

### 2.2 Basal Ganglia and Action Selection - Role of Striatal Input

It has been suggested that the basal ganglia provide the vertebrate brain with a solution to a selection problem that arises whenever two or more competing functional neural systems (*e.g*., subserving energy balance and defence in the context of the present review) seek simultaneous access to a restricted motor resource (behavioural output) [1]. Considering several computational selections, architectures suggest significant advantages for systems incorporating a central switching mechanism. Therefore, it has been proposed that the vertebrate basal ganglia have evolved as a centralized selection device, specialized in resolving conflicts over access to limited motor resources (and, by extension, cognitive and emotional resources) [1]. Analysis of basal ganglia functional architecture and its position within a wider anatomical framework suggests it can satisfy many of the requirements expected of an efficient selection mechanism.

Current views of information processing within the basal ganglia are heavily influenced by the suggestion of “multiple parallel channels” *e.g*., [8]. These channels originate in the cerebral cortex, project *via* topographically segregated pathways to the striatum, through the basal ganglia nuclei, and return *via* a thalamic relay to the cortex region from which the specific corticostriatal projections originated. The model assumes that the internal circuitry of the basal ganglia is configured so that, at the level of output nuclei (*e.g*., the substantia nigra pars reticulata), selected channels are inhibited while non-selected channels are excited. Because of the inhibitory nature of the basal ganglia output, targets of selected channels will be disinhibited while inhibition on non-selected channels is maintained or increased. In this manner, activity in the most actively supported (salient) functional system will be sustained, and its direct link with the motor systems will be unblocked. Conversely, less well-supported functional systems will be inhibited, and their access to the motor systems will be denied. In other words, the functional system (*i.e*., hunger, thirst or defense) with the most salient sensory and contextual inputs will have its channels selected in the basal ganglia and its outputs selectively disinhibited. Consequently, the problem of access to restricted behavioral resources will be resolved on a winner-take-all basis. This idea has been extended by suggesting that the basal ganglia can decide between competing systems, not only represented in the cerebral cortex but also between those in the evolutionarily older brainstem [5]. Based mainly on anatomical evidence, we suggested that many subcortical structures that can guide movements, such as the superior colliculus, the parabrachial nucleus or the periaqueductal grey nucleus, also have connections with the basal ganglia that may represent a series of parallel, at least partially closed-loops. In contrast with the cortico-basal ganglia loops, the subcortical loops have a thalamic relay on the input rather than on the return-link of the circuit.

### 2.3 Basal Ganglia and Action Selection - Role of Dopaminergic Input

Dopaminergic neurons located in the substantia nigra pars compacta, and the ventral tegmental area are sensitive to unexpected, biologically salient stimuli, including those associated with reward [17]. If the presentation of a reward is reliably predicted by another stimulus, such as a light or a tone, the burst of activity gradually transfers from the reward to the predicted stimulus. Once a conditioned response has been established and the predicting stimulus is not followed by the expected reward, a reliable depression in the spontaneous activity of the dopaminergic neurons is observed shortly after the expected reward delivery. It has been suggested that these dopaminergic bursts or pauses are respectively signaling when something better or worse than expected is occurring, representing a reward prediction error [18]. More recently, a new hypothesis further developed this idea and proposes that rather than directly reinforcing responses that lead to reward in the Thorndike ‘Law of effect’ sense as implied by the concept of reward prediction error, the short-latency dopamine signal enables the basal ganglia to discriminate unexpected sensory events in the world that may have been caused by actions of the agent from those resulting from other sources [19]. Indeed, through behavioural repetition, the basal ganglia may assist in developing entirely new actions with specific positive or negative sensory outcomes. Phasic dopamine signals reinforce the reselection of actions that immediately precede an unpredicted biologically salient event (phasic dopamine bursts) or dismiss the selection of actions that immediately precede a negative outcome (phasic dopamine inhibition). This form of learning in the basal ganglia may be a critical part of the mechanism by which the brain accumulates a ‘library’ of action-outcome associations, which, on later occasions, can be dynamically configured to select actions / behavioral sequences leading to the best rewarding effect.

### 2.4 Basal Ganglia and Action Selection - Role of the Subthalamic Nucleus Input

As described previously, the subthalamic nucleus is also a major input structure of the basal ganglia. In the context of the action-selection model, it has been suggested that rapid control of the subthalamic nucleus *via* the hyper-direct pathway is of great importance [20]. Indeed, it should be assumed that once the most salient action has been selected, it will remain in competition with more bids entering from other functional brain systems, and one of them may become the most salient one. Therefore, to be able to switch from the old selected action to the new one, the ongoing action must first be halted before allowing the new action to start. The subthalamic nucleus seems to fulfill all the requirements for this purpose [16]. The structure receives widespread connections from many brain regions, including cortical motor areas and the thalamus, but also from subcortical sensorimotor structures such as the superior colliculus, the parabrachial nucleus and the periaqueductal grey nucleus [21-24]. The evidence reviewed by Mink [10] and Smith [25] suggests that corollary signals directed to the subthalamic nucleus produce a rapid and diffuse excitation of the basal ganglia output nuclei prior to the arrival of more focused disinhibitory signals from the striatum [26, 27]. This temporary excitatory effect on the output nuclei could dispel the disinhibitory activity associated with current selections and send a brief wave of inhibition to the brainstem and thalamic targets of the basal ganglia. Therefore, the outcome would be to interrupt or pause ongoing actions and establish circumstances in which a new selection may be more easily imposed. In the context of this review, an animal engaged in an approach behavior for feeding should cease this action in response to an attack by a predator to select a more appropriate defensive behavior from inputs/bids to the striatum.

### 2.5 Basal Ganglia and Action Selection - Summary

The basal ganglia have the key function of directing our behavior in the context of events from our environment and/or our internal state. We have seen that this function relies on afferents targeting the main input structures of the basal ganglia, entering bids for action selection at the level of the striatum or signals for behavioral interruption at the level of the subthalamic nucleus, with behavioral reselection facilitated by dopamine signaling. In the next sections, we will describe the current knowledge concerning how brainstem structures such as the superior colliculus, the parabrachial nucleus and the periaqueductal grey nucleus project to these main input nuclei of the basal ganglia to explore the functional consequences of those interactions in the context of approach/avoidance behavior.

## 3 THE SUPERIOR COLLICULUS

### 3.1 Functional Anatomy of the Superior Colliculus

The superior colliculus is one of the most well-studied brainstem structures. The colliculus has a layered organization due to the distribution of the fibers and variation in the size and density of neurons [28]. It is composed of seven layers that are grouped into two divisions. The dorsal superficial layers are almost exclusively visual and receive visual inputs directly from retinal ganglion cells [29-30] and indirectly from the striate cortex [31]. The ventral intermediate and deep layers are multi-modal and process visual signals from the superficial layers as well as somatosensory and auditory input [32]. Both divisions contain a topographic map of the visual field but neurons from the intermediate and deep layers have wider receptive fields [32]. For both divisions, the upper field is represented medially in the colliculus, from which avoidance/escape responses are elicited. The lower field is represented laterally, from where orienting responses are elicited [29, 33-35]. In rats and mice, unexpected stimuli appearing in the upper visual field are likely to be predators, and therefore avoidance/escape is the most adaptive reflexive behavioral response. In contrast, unexpected stimuli in the lower visual field are more likely to be food or offspring; hence, the approach is the most adaptive reflexive behavioral response.

In the primate, extensive literature associates the deep and intermediate layers of the superior colliculus with orienting of the head and eyes [36-40]. However, microinjection of the GABA_A_ antagonist bicuculline into the intermediate and deep layers of the colliculus in macaques has been reported to elicit cowering, escape-like behavior, high-pitched vocalization, and attack of objects [41]. Although the authors report that responses could be elicited from both medial and lateral aspects of the structure [41], the spread of injection was reasonably large (3 mm diameter at 1 hour) *versus* the width of the macaque colliculus (~5 mm), which leaves open the possibility of inclusion of the medial colliculus in most if not all injections. That said, the ecological niche of the macaque is very different from the rat and mouse, so functional specialization in the mediolateral axis may not be relevant. Although avoidance/escape responses have not been explicitly reported following collicular stimulation in other species, the colliculus in a wide range of species contains neurons sensitive to looming stimuli (pigeon optic tectum, [42], frog optic tectum, [43], mouse, [44], cat, [45], humans, [46]), which indirectly suggests a role in avoidance/escape.

Approach and avoidance/escape at the level of the superior colliculus are modulated by differential inputs to the lateral and medial areas in the rat [47]. Small injections of the retrograde tracer Fluorogold into the medial or lateral areas of the intermediate and deep layers of the colliculus revealed that there are a number of structures that only project to the medial (*e.g*., retrosplenial cortex, temporal association cortex, lateral geniculate and supra geniculate thalamic nuclei, ventromedial and pre mammillary hypothalamic nuclei) or lateral (*e.g*., primary somatosensory cortex representing upper body parts and vibrissae and parvocellular reticular nucleus in the brainstem) colliculus. Other structures were found to project both medial and lateral areas but from topographically segregated populations of neurons (*e.g*., zona incerta and substantia nigra pars reticulata).

As well as segregation at the afferent level, in rats, cells in the intermediate and deep colliculus give rise to segregated outputs – the lateral intermediate/deep layers give rise to projections that cross midline (the ‘contralateral descending pathway’), while the medial intermediate/ deep layers give rise to projections that do not cross the midline (the ‘ipsilateral descending pathway’, [48]. The contralateral descending pathway projects caudally *via* the predorsal bundle to the contralateral medial pontomedullary reticular formation and upper cervical spinal cord. The ipsilateral descending pathway projects to the cuneiform nucleus and ventrolateral pontomedullary reticular formation [49, 50]. In addition to the descending projections of the superior colliculus, it is now clear that the lateral and medial intermediate and deep layers also send non-overlapping ascending projections to areas of the thalamus, which in turn project differentially to the striatum (see below).

### 3.2 Subcortical Loop between the Superior Colliculus and the Basal Ganglia

The subcortical loops described earlier have been characterized at the level of the superior colliculus by McHaffie and colleagues [5] according to the superficial/intermediate and deep layers subdivisions. Here we will describe how loops can also be found based on the other major functional subdivision of the superior colliculus into medial/lateral aspects involved in avoidance/approach, respectively.

Tract tracing studies using anterograde tracers have confirmed that the intermediate and deep layers of the superior colliculus project to the parafascicular, rostral intralaminar (the central lateral, paracentral and central medial nuclei) and ventromedial nuclei of the thalamus [51]. However, more detailed investigations indicate a more specific organization from the colliculus to the thalamus when considering its lateral and medial aspects. In summary, evidence suggests that efferents from the lateral and medial aspects of the colliculus form part of independent loops that traverse the thalamus, striatum and substantia nigra pars reticulata, returning to the colliculus *via* the nigro-tectal projection to complete the loops. We will now consider those loops in more detail using retrograde tracers [52], the parafascicular, rostral intralaminar and ventromedial nuclei of the thalamus all receive input from the lateral portion of the ipsilateral colliculus, primarily from the intermediate layers. These projections have also been confirmed electrophysiologically. Yamasaki and Krauthammer [53] antidromically activated cells in the intermediate and deep layers of the superior colliculus by stimulation of the parafascicular nucleus and centrolateral nucleus in the rat. We have shown that anterograde labeling of thalamic fibers is extensive in the striatum following BDA injections into the rostral intralaminar nuclei [54]. Staining is seen primarily in the mid to lateral regions of the anterior striatum. Striatal staining is also extensive following injections into the parafascicular nucleus, with labeling seen throughout the rostrocaudal extent of the striatum. These findings echo those reported by Pan *et al.* [55] and Schwab *et al.* [56]. In the rat, clear, dense patches of fibers are also observed in the lateral regions of the striatum as a result of injections into the ventromedial thalamic nucleus, contrasting with the preferential projection into the medial striatal regions in the mouse [55]. So, evidence is clear that the lateral colliculus in the rat projects through the parafascicular nucleus, rostral intralaminar nuclei and the ventromedial nucleus to the striatum.

Our own work [54] in the rat has confirmed previous reports of thalamic projections from the lateral colliculus and extended these findings to the medial aspect of the structure. Medial injections of an anterograde tracer result in very light staining in the dorsal parafascicular nucleus and dorsal rostral intralaminar nuclei. We have confirmed these anterograde findings by iontophoretic administration of the retrograde tracer Fluorogold. This clearly suggests that the medial colliculus may have other thalamic targets to reach the striatum. We have found that injections of anterograde tracer into the intermediate and deep layers of the medial colliculus label the posterior intralaminar thalamic nucleus [54]. Iontophoretic injection of Fluorogold into the posterior intralaminar thalamic nucleus gives rise to a large amount of retrograde labelling in the medial colliculus with the densest patches in the medial intermediate and deep layers. In terms of projections forward to the striatum, injections of the retrograde tracer Fluorogold into the caudal striatum label the posterior intralaminar thalamic nucleus, whereas injections into the rostral striatum do not. Anterograde labeling in the striatum from an iontophoretic injection of BDA into the posterior intralaminar thalamic nucleus confirmed a projection to the very caudal tail of the striatum [57, 58], suggested to be processing specific aversive signals [59]. The caudal striatum onto which the medial colliculus is projecting appears to be an area that is structurally and functionally separate from the rest of the striatum. Here, there appears to be a substance-P rich band where medium spiny projection neurons almost exclusively express D1-type dopamine receptors and an enkephalin/substance-P poor band where medium spiny projection neurons almost exclusively express D2-type dopamine receptors [60, 61]. Experiments in the monkey suggest that this area of the striatum (the ‘tail’ of the striatum in the monkey) has different functions than the head of the striatum [62, 63]. Interestingly, in keeping with a functional focus on avoidance/escape, dopaminergic neurons projecting to the tail do not encode reward value but instead, appear to play a role in the avoidance of threatening stimuli [64]. Furthermore, optogenetic inhibition of the caudal striatum blocks looming-induced freezing in mice [65].

The caudal striatum projects to the lateral edge of the zona reticulata region of the substantia nigra and the pars lateralis [66]. Cells of origin of the nigrotectal tract in the pars lateralis project to the medial colliculus [67], at least partially closing the loop. The extensive areas of the striatum that form the projection target of the parafascicular nucleus, rostral intralaminar nuclei and the ventromedial nucleus innervate widespread areas of the reticulata [66], which in turn projects to the lateral colliculus [67], again at least partially closing the loop. The pathways linking the medial and lateral colliculus to the thalamus and striatum are illustrated in Fig. (**1A**).

In parallel with the action selection bids from the superior colliculus to the striatum routed through the thalamus, the colliculus also interacts with the basal ganglia in two other important ways: *via* dopaminergic neurons in the substantia nigra pars compacta (SNc) and ventral tegmental area (VTA), and the subthalamic nucleus. Unlike the projections operating *via* the thalamus, the projections to the SNc and the subthalamic nucleus (at least in the rat) are from a more restricted area of the colliculus.

### 3.3 Superior Colliculus Projections to Dopaminergic Neurons

Our group has revealed a direct projection from the superior colliculus to the SNc and the VTA, which we have shown to relay visual information to these structures [68-70]. We have also shown that this pathway is complex, involving presumed excitatory (the majority) and inhibitory (the minority) components. It provides afferents to both dopaminergic and non-dopaminergic neurons in the area [69], which may underlie excitatory and inhibitory responses in dopaminergic neurons in both the SNc and the VTA [70]. While not highlighted in our previous publications, following injections of anterograde tracers such as PHA-L or BDA into the colliculus, terminals within the SNc were much more numerous when the tracers were injected in a specific subregion of the superior colliculus located in the deep lateral part of this structure [69].

The existence of the ‘tectorial pathway’ and ‘tecto-VTA’ pathway has been confirmed in other species, such as mice [71, 72], cats [73] and primates [74], with cells of origin in the deep and intermediate layers of the superior colliculus. Interestingly, a detailed investigation of these pathways made by Huang and colleagues [71] showed that the majority of superior colliculus cells projecting to the dopaminergic neurons are glutamatergic (~ 92%) with some GABAergic cells (GAD2+ cells), and that specific activation of these pathways using optogenetics triggers dopamine release in the striatum. Furthermore, specific modulation of the tectonigral pathway using optogenetics favors orienting behavior in mice, regulating appetitive locomotion in predatory hunting without affecting the avoidance behavior [71]. In terms of the dichotomy between approach and avoidance/escape, activating the tecto-VTA pathway favors orienting responses, and a long-lasting phasic activation of the tecto-VTA pathway promotes head/body movements [72]. Overall, these anatomical and behavioral results suggest that the tecto-nigral and tecto-VTA pathways mainly subserve approach/ appetitive functions, as might be anticipated, given their origin in the lateral part of the colliculus.

It has been suggested that phasic dopamine signals have a role in discovering novel actions [19]. The work from Vautrelle and colleagues [75] presented in this special issue highlights the role of the visual input from the colliculus to dopaminergic neurons in potentiating motor signals arriving at the striatum. Visually-induced dopamine release in the striatum following a motor action has been suggested to have a reinforcing function [76]. Since this dopamine release is generated by a pathway that is mainly involved in the transmission of approach or appetitive signals, it is likely that the function of collicular-induced dopamine release mainly subserves a positive reinforcement function. The pathways linking the medial and lateral colliculus to the SNc and VTA are illustrated in Fig. (**1B**).

### 3.4 Superior Colliculus Projections to the Subthalamic Nucleus

Tokuno and colleagues [21] were the first groups to raise the issue that many studies were centered on the role of the output nuclei of the basal ganglia in the control of the superior colliculus, but none seemed to have paid attention to the direct influence the colliculus may have on the basal ganglia. Using anterograde and retrograde tract-tracing neuroanatomy, they described a direct projection between the colliculus and the subthalamic nucleus arising mainly from the middle to caudal parts of the deep layers and terminating in the dorsal part of the subthalamic nucleus. In this work, the tecto-subthalamic pathway did not appear to provide a particularly strong input to the subthalamic nucleus. However, whilst investigating the tecto-nigral and tecto-VTA pathways described above, we noticed the presence of a denser layer of terminals in the dorsal part of the subthalamic nucleus previously described by Tokuno and colleagues [21]. We then characterized the topography of this pathway and found that in order to label the pathway, the anterograde tracer has to be injected in the very lateral part of the intermediate and deep layers of the colliculus to produce the densest terminal labeling in the subthalamic nucleus [22]. We also went on to show that this pathway is largely excitatory since collicular terminals make asymmetric synaptic contacts within the dorsal and rostral part of the subthalamic nucleus, and activation of this pathway using visual stimuli has an excitatory effect on subthalamic cells [22]. This pathway has recently been confirmed to be a bilateral projection in the rat [24].

In the context of the action-selection model previously described, as has already been mentioned, the rapid control of the subthalamic nucleus by afferent structures is important [20]. In that regard, the link between the superior colliculus and the subthalamic nucleus is likely to be of particular functional significance. The colliculus can be compared to a “novelty detector”: any unexpected event happening in the environment is likely to phasically activate this structure at a short latency [77]. The transmission of this information to the subthalamic nucleus is crucial as those novel events may be of vital importance to the animal and require a change of behavioural output in response to them. Similar to the tecto-nigral and tecto-VTA pathways, the cells of the origin of the text-subthalamic pathway are also localized in the very lateral part of the intermediate and deep layers of the colliculus. While to date, no specific optogenetic study has been conducted manipulating this pathway to fully evaluate its behavioral effects, given the position of the cells of origin in the colliculus and the functions ascribed to its lateral aspect, the visual signals to the subthalamic nucleus are likely to operate within appetitive/approach context. The pathways linking the medial and lateral colliculus to the subthalamic nucleus are illustrated in Fig. (**1C**).

### 3.5 Links between the Superior Colliculus and the Basal Ganglia - Summary

In summary, the superior colliculus is in a position to contribute to basal ganglia action selection *via* inputs that subserve approach and avoidance/escape functions. This is partially achieved *via* projections to the thalamus, which then relays information to the striatum. The striatum then, in turn, projects to the substantia nigra pars reticulata and then back to the colliculus in what has been described as subcortical loops [5]. These collicular projections to the thalamus cover both approach and avoidance/escape. However, other inputs to the basal ganglia, from the colliculus to the dopaminergic neurons of the ventral midbrain and the subthalamic nucleus, are more selective. The SNc/VTA and subthalamic nucleus receive signals from the approach-specific part of the colliculus, at least in the rat. What this suggests is that fast avoidance/escape signals may be provided by other brainstem structures. According to the literature, we identified two other candidates processing signals relevant to avoidance/escape, which share similar connectivity with the basal ganglia as the colliculus, namely the parabrachial nucleus and the periaqueductal grey nucleus.

## 4 THE PARABRACHIAL NUCLEUS

### 4.1 Functional Anatomy of the Parabrachial Nucleus

The parabrachial nucleus is located in the dorsolateral pons and surrounds the superior cerebellar peduncle as it enters the brainstem from the cerebellum [78, 79]. Until the end of the 80s, the parabrachial nucleus was known as the gustative pontine nucleus [80, 81]. It has since been shown to be involved in hunger, cardio-vascular regulation, respiration, wakefulness control and thermoregulation [82-87]. The parabrachial nucleus is, therefore, a central structure supporting the organism’s general homeostasis [88, 89]. Importantly in the present context, the parabrachial nucleus also generates autonomic responses following the occurrence of painful stimuli [90, 91] and processes nociceptive information [92-94]. This structure is a major target for nociceptive neurons located in layers I-II in the spinal cord. In the cat, it has been estimated that 6000 spinal neurons project to the parabrachial nucleus in comparison to 1500 neurons for the spinothalamic tract [95]. Spinal neurons send a bilateral projection to the lateral part of the parabrachial nucleus, with a predominance to the contralateral side [96]. Pain clearly plays a role in both avoidance and escape.

The lateral parabrachial nucleus has been shown to be rich in neurons positive for the neurochemical marker calcitonin gene-related peptide (CGRP), which is usually found in visceral nociceptive pathways [89]. Through the projections of these neurons to limbic structures such as the amygdala, hypothalamus, and insular cortex, CGRP neurons from the lateral parabrachial nucleus have a crucial role in the emotional component of pain [94, 97-99]. CGRP neurons in the parabrachial nucleus have been suggested to function as a general alarm system to maintain homeostasis, with pain being one of the most important contexts [89, 100]. Accordingly, it has been shown that CGRP neurons in the parabrachial nucleus are involved in the development of aversive and defense behavior [101-103] but are poorly involved in the precise location of the painful stimuli due to their large receptive fields [104]. In addition, tachykinin 1 positive neurons in this structure have also been found to modulate nocifensive behaviors [102, 105]. Overall, the parabrachial nucleus is a structure that can to encode the presence of something dangerous and promote affective behavioral states that limit the harm in response to potential threats [106]. Indeed, it has been recognized as a hub for pain and aversion [107]. This subcortical structure could contribute to basal ganglia action selection by providing selective avoidance/escape bids for expression that parallel the selective approach-related inputs to the SNc and subthalamic nucleus provided by the superior colliculus.

### 4.2 Subcortical Loop between the Parabrachial Nucleus and the Basal Ganglia

The subcortical loops described by McHaffie and colleagues [5] mainly involved the superior colliculus. However, the authors suggested that other midbrain and hindbrain structures, including the parabrachial nucleus or the periaqueductal gray nucleus, also have the potential to provide input to the striatum and hence the basal ganglia *via* a relay in the midline intralaminar complex of the thalamus. Similar to the superior colliculus, the parabrachial nucleus is also subdivided into different subnuclei, each with its own unique set of efferent projections, especially toward the thalamus, which may thus represent different channels toward the striatum [108-110]. The parabrachial nucleus can be broadly divided into two main regions, the lateral and the medial parabrachial, according to their afferent inputs. The medial parabrachial has been implicated in gustatory functions *via* its innervation from the gustatory region of the nucleus of the solitary tract and cerebral cortex [111], while, as seen previously, the lateral parabrachial is generally involved in nociception through nociceptive input from the spinal cord, the visceral fibers from the nucleus of the solitary tract and the amygdala [91, 112-114]. Both regions preferentially project to different thalamic areas. The medial parabrachial nucleus densely innervates areas of the intralaminar thalamus, such as the lateral and medial part of the parafascicular nucleus, while the lateral parabrachial nucleus densely innervates the midline thalamic area, such as the paraventricular and inter mediodorsal nuclei [110]. The paracentral thalamic nucleus is the exclusive target of a specific subnucleus located in the dorsal region of the lateral parabrachial, the internal lateral parabrachial nucleus, which conveys and encodes cutaneous nociceptive information from layer V/VI of the spinal cord [115, 116]. More recently, ascending pathways from the lateral and medial parabrachial nucleus have been further analyzed according to their molecular identity [117]. The authors have shown the diversity of subclusters of neurons within each subregion of the PBN with specific patterns of projections [117]. As previously discussed in relation to the superior colliculus, these thalamic nuclei are known to then project to the striatum. The parabrachial nucleus is also a major target of the basal ganglia output structures, at least the substantia nigra pars reticulata (SNr) [13-118], closing the parabrachial - basal ganglia loop. Unlike the superior colliculus, the functional link between the basal ganglia and the parabrachial nucleus in the context of taste-guided behavior, such as conditioned taste aversion/preference or response to potential threats, to our knowledge, has never been considered and studied. The pathways linking the parabrachial nucleus to the thalamus and striatum are illustrated in Fig. (**2A**).

### 4.3 Parabrachial Nucleus Projections to Dopaminergic Neurons

Experiments on SNc/VTA dopaminergic neurons classically report that these cells exhibit a short latency, short duration phasic excitation to unpredicted stimuli that are salient through their novelty and intensity or their reward value [119-121]. Importantly, in various mammals, they also exhibit a short latency phasic decrease of activity when an expected reward fails to appear or in response to noxious/aversive stimuli [121-124]. Our group raised the fact that while much was known about many aspects of the ascending dopamine systems, surprisingly, little was known about the sensory inputs that phasically modulate their activity [123]. Our tract tracing neuroanatomy experiments revealed a direct projection between the parabrachial nucleus and the SNc/VTA. We found that the general distribution of retrogradely labelled cells in the parabrachial nucleus was similar following an injection of a retrograde tracer in the lateral and central part of the SNc or the VTA, suggesting that the projection appears to innervate the whole dopamine-containing region. These parabrachial – SNc/VTA cells were found in all subnuclei of both the lateral and medial parts of the parabrachial nucleus, with no clear presence of a topographical organization. However, a significantly greater density of cells was labelled in the nociceptive lateral part of the parabrachial nucleus compared to the medial part, especially within the rostral and caudal areas of this structure [125].

We have also shown that inactivating the parabrachial nucleus pharmacologically attenuates, and in some cases eliminates, nociceptive responses of dopaminergic neurons located in the SNc [125]. Altogether, we have demonstrated that the parabrachial nucleus is a substantial source of nociceptive signals to midbrain dopaminergic neurons. Although the existence of a pathway linking the parabrachial nucleus to the SNc/VTA has subsequently been confirmed [126-128], our results raised two intriguing issues. The first is the fact that activating parabrachial cells generates an inhibitory response in dopaminergic neurons, although parabrachial projecting neurons are unlikely to be GABAergic in nature. Indeed, the lateral parabrachial – VTA projection seems to mainly consist of glutamatergic neurons [126-128]. The second is that the delay between parabrachial activation and dopaminergic inhibition appears long (~ 50 ms), especially in the context of nociception (mean ± SEM: 9.2 ± 0.4 ms for activation of the parabrachial nucleus *vs.* 58.7 ± 2.9 ms for activation of cells in the VTA). Some light has been cast on these issues by recent studies. Yang and colleagues [127] have suggested that neurons from the lateral parabrachial nucleus indirectly inhibit VTA dopaminergic neurons by activating GABAergic neurons in the VTA, which may explain the delay between the parabrachial and SNc/VTA responses. They studied the functional connectivity between the lateral parabrachial nucleus and the dopaminergic / GABAergic cells in the VTA as well as GABAergic cells in the SNr. They have found that optogenetic excitation of the parabrachial-VTA glutamatergic pathway induced large excitatory post-synaptic currents in tyrosine hydroxylase (TH)-positive, presumed dopaminergic neurons and TH negative/vesicular GABA transporter-positive neurons in the SNr, but not in GABAergic neurons in the VTA.

A direct projection between the parabrachial nucleus and the SNr had previously been described in the rat [13] and further studied by Yang and colleagues in mice [127] in order to understand its role in the inhibitory response of VTA dopaminergic neurons following parabrachial activation. SNr neurons are mainly excited at short latency by noxious stimulation, which will exert an inhibitory influence on parabrachial neurons projecting to the VTA. Yang *et al.* [127] hypothesized that inhibiting parabrachial neurons targeted by the SNr in response to noxious stimuli inhibits VTA dopaminergic neurons through a feed-forward inhibition between the parabrachial nucleus and the SNr. However, this indirect inhibition may also involve the projection between the parabrachial nucleus and the rostromedial tegmental nucleus (RMTg)/tail of the VTA [129], a unique GABAergic afferent to the midbrain dopaminergic neurons connected to numerous structures involved in aversive learning and punishment, including the parabrachial nucleus. It has been shown that the parabrachial nucleus projection to the RMTg/tail of the VTA specifically transmits information about the punishment signals (shocks) in punishment learning [130], which may then inhibit the activity of dopaminergic neurons.

Overall, these results suggest that the inhibition of dopaminergic neurons observed following a negative event is probably indirectly generated by nociceptive/aversive structures. Why such an indirect anatomical circuit underlies, this inhibition remains to be fully understood. Note that another inhibitory input from the dorsal raphe nucleus should be considered here. Stimulation of this structure, hence increased release of 5-HT at the level of the nigrostriatal system, has been shown to slowly inhibit dopaminergic neurons [131]. The role of the direct glutamatergic parabrachial-VTA/SNc pathways also remains to be fully elucidated, although Zhang and colleagues [128] have recently linked this pathway to the development of depressive symptoms in chronic neuropathic pain in mice in which the parabrachial nucleus is strongly involved [132]. The pathways linking the parabrachial nucleus to the SNc and VTA are illustrated in Fig. (**2B**).

### 4.4 Parabrachial Nucleus Projections to Subthalamic Nucleus

Given the similarities between the projections from the superior colliculus and parabrachial nucleus to the striatum and the ventral midbrain, it was likely that this structure would (like the colliculus) also be anatomically linked to the subthalamic nucleus. The first evidence of such projection can be seen in the literature studying the efferent projections of the parabrachial nucleus to its more classical targets, including the thalamus, the hypothalamus and the amygdala [133], or [134], Fig. (**2C**). We have confirmed the existence of this pathway between the parabrachial nucleus and the subthalamic nucleus [23]. Our injection of an anterograde tracer into the parabrachial nucleus revealed a robust direct projection to the ipsilateral subthalamic nucleus and a less substantial projection to the contralateral side. The parabrachial terminals were differentially distributed within sub-regions of the subthalamic nucleus and largely seen in a dorsal sheet that extended across the entire mediolateral axis of the structure, covering the dorsoventral portion at a more rostral level. The location of the projection in the subthalamic nucleus is similar to the one described previously for the tecto-subthalamic pathway. The injection of a retrograde tracer in the subthalamic nucleus labeled numerous cells in the parabrachial nucleus, supporting the presence of this pathway. Similar to the parabrachial-nigral pathway, retrogradely labeled neurons were found in all subnuclei of both the contralateral and ipsilateral parabrachial nucleus, including within the fibers of the superior cerebellar peduncle. However, the ipsilateral side had a significantly greater cell density compared to the contralateral side. The largest density of cells projecting to the subthalamic nucleus from the lateral parabrachial was located in the rostral part of this structure, while the majority of the medial parabrachial cells projecting to the subthalamic nucleus were found in the posterior parabrachial nucleus. We have also demonstrated that the parabrachial nucleus is a crucial relay for nociceptive signals directed at the subthalamic nucleus and suggested that this projection was part of a novel nociceptive network linking the parabrachial nucleus to the basal ganglia [23]. As discussed previously [23-135], this projection is consistent with the presumed role of the subthalamic nucleus in the interruption of ongoing behavior [136] in the context of the role of the basal ganglia in action selection [1]. Nociceptive signals transmitted by the parabrachial nucleus to the subthalamic nucleus clearly indicate that behaviour with negative consequences is taking place and hence needs to cease. However, with the involvement of the parabrachial nucleus in chronic pain, we also suggest that this pathway may have a wider function, similar to the role of the parabrachial - dopaminergic pathway previously described [132]. The pathways linking the parabrachial nucleus to the subthalamic nucleus are illustrated in Fig. (**2C**).

## 5 THE PERIAQUEDUCTAL GREY NUCLEUS

### 5.1 Functional Anatomy of the Periaqueductal Grey Nucleus

The periaqueductal grey nucleus is the portion of the ventricular gray matter surrounding the midbrain aqueduct. There is an agreement that this structure is involved in the coordination of survival and emotional defensive behaviors through its subdivision into four columns along its rostrocaudal axes, the dorsomedial (dmPAG), dorsolateral (dlPAG), lateral (lPAG) and ventrolateral columns (vlPAG) [137, 138]. The periaqueductal grey nucleus plays a major role in integrating responses to internal and external threats that maximize an animal’s survival by generating a repertoire of conditioned and unconditioned fear behavior [139, 140]. The dmPAG, dlPAG and lPAG have been shown to be involved in orchestrating active coping strategies such as fight or flight when presented with a threat [137, 141] and the vlPAG in passive coping strategies such as freezing, occurring, for example, to avoid detection [142, 143]. The behavioral sequence generated by each column varies according to the rostrocaudal level in the periaqueductal grey nucleus [139]. Furthermore, a more recent experiment demonstrated that the link between column and behavioral output is more complicated when considering genetically-defined populations of neurons within this structure. Specific activation or inhibition of cholecystokinin-expressing cells in the lPAG and vlPAG can cause a flight to safer regions or reduce predator avoidance without altering other defensive behavior like freezing [144]. The periaqueductal grey nucleus is also implicated in a wide range of physiological functions, probably to optimize these active and passive behavioral sequences, including respiration, cardiovascular changes, vocalization, and pain processing [145-149]. The vlPAG has been shown to be a major site of endogenous opioid-induced pain suppression, and electrical stimulation of the vlPAG produces profound analgesia [143, 150]. It has been suggested that glutamatergic projections from the periaqueductal grey nucleus exert antinociceptive effects, whereas GABAergic projections exert pronociceptive effects on pain transmission through descending pathways [151].

As reviewed by Silva and McNaughton [140], the periaqueductal grey nucleus is strongly interconnected with numerous cortical, subcortical and spinal areas and is positioned at the interface between limbic structures (amygdala, hypothalamus, medial prefrontal cortex) and the lower brainstem (superior colliculus, parabrachial nucleus, raphe nucleus), hence within a circuit essential to survival, whose role and organization has been extensively discussed *i.e*., [139, 140, 152-154]. The role of the periaqueductal grey nucleus has mainly been studied in relation to the circuit involved in survival and emotional defensive behaviors, especially involving the amygdala. The functional and anatomical links toward the basal ganglia have been sparsely described and discussed. In this review, we introduce the possibility that the periaqueductal grey nucleus shares the same connectivity with the basal ganglia as the superior colliculus and the parabrachial nucleus. Accordingly, this subcortical structure could also contribute to the role of the basal ganglia in action selection and provide different anatomical channels to favor particular kinds of defensive/survival responses, again consistent with a role in the broad functionality of avoidance/ escape.

### 5.2 Subcortical Loop between the Periaqueductal Grey NUCLEUS and the Basal Ganglia

Similar to the parabrachial nucleus, Krout and Loewy [155] provided the first exhaustive analysis of periaqueductal grey nucleus projections to the midline and intralaminar thalamic nuclei in the rat, which in turn are known to project to the striatum [156, 157]. This anatomical analysis was performed according to the classical idea that the periaqueductal grey nucleus is divided into 4 columns. This work showed that the number of periaqueductal-thalamic fibers increases across the dorsoventral axis, from small numbers arising from the dorsal part of the periaqueductal grey nucleus to dense projections arising from the ventral part. An anterograde tracer injected into the dmPAG labeled a small number of axons/terminals, specifically within the paraventricular thalamus. An injection into the dlPAG column gave rise to a similar axonal/terminal distribution as the dmPAG but with more intense labeling. In contrast, injections into the lPAG densely labeled each of the intralaminar thalamic nuclei (except the parafascicular nucleus). Finally, the vlPAG projection to the thalamus was the heaviest of all, terminating densely in the central medial thalamus and moderately in the paraventricular thalamus. Krout and Loewy’s [155] corresponding retrograde experiment, in which a retrograde tracer was injected into different parts of the midline and intralaminar thalamus, further highlighted the importance of the rostrocaudal axis of the periaqueductal grey nucleus in terms of the organization of the periaqueductal-thalamic pathways. For example, the intermediate rostrocaudal level of the dlPAG heavily targets the anterior paraventricular thalamus, while the most caudal part of the vlPAG targets the paraventricular more compared to other levels of the periaqueductal grey nucleus. Overall, while a more refined characterization is now needed to reconstruct the specific topography of the periaqueductal-thalamic projections in 3 dimensions, a general channel-like organization stands out with the dorsal periaqueductal grey nucleus having the densest (but not exclusive) projection to the paraventricular nucleus, the lPAG to the intralaminar nuclei and the vlPAG to the central medial thalamus. Again, the output structures of the basal ganglia close the loop with a direct projection between the SNr and the periaqueductal grey nucleus [6, 118], which we have shown to target the entire periaqueductal grey nucleus in our preliminary work in the rat, but with the strongest projection to the vlPAG.

A channel-like functional organization between the dlPAG and the thalamus has recently been studied in the context of fear learning using optogenetic modulation and viral tracing approaches [153]. The authors discovered that the dlPAG but not the vlPAG was important for acquiring aversive memories. They also found that a population of neurons in the dlPAG projecting to the anterior paraventricular nucleus was specifically involved in this acquisition, but dlPAG cells projecting to the posterior paraventricular nucleus or the central medial nucleus were not. Finally, they found that the dlPAG-anterior paraventricular cells do not collateralize to the posterior paraventricular or central medial nucleus of the thalamus. They thus identified a population of cells in the dlPAG that project to the anterior paraventricular nucleus that is involved in fear memory formation. Similar to the parabrachial nucleus, the function of the periaqueductal grey-recipient thalamic projections to the striatum has not been considered or studied to our knowledge. The pathways linking the periaqueductal grey nucleus to the thalamus and striatum are illustrated in Fig. (**3A**).

### 5.3 Periaqueductal Grey Nucleus Projections to Dopaminergic Neurons and THE Subthalamic Nucleus

The projection from the periaqueductal grey nucleus to dopaminergic neurons has mainly been studied in relation to the VTA [158-160] as part of its involvement in nociceptive modulation [161, 162] and the expression of fear and aversive responses [163-165], in which the periaqueductal grey nucleus is also strongly involved. The periaqueductal grey nucleus sends a bilateral projection to the VTA, partly *via* glutamatergic neurons (VGLUT2+) densely localized in the ipsilateral vlPAG [160]. However, the cells involved in the periaqueductal-VTA pathway are not only glutamatergic [160]. They do not appear to be catecholaminergic (dopaminergic or noradrenergic), at least from the vlPAG [166], which suggests the possible involvement of a GABAergic projection from the periaqueductal grey nucleus to the VTA. Similar to tecto-nigral neurons, periaqueductal grey neurons synapse directly onto dopaminergic and GABAergic neurons in the VTA [165, 167], and both symmetric and asymmetric synapses have been described in these two neuronal populations in this structure [167]. This suggests that the periaqueductal grey nucleus is in a position to excite dopaminergic neurons directly or indirectly *via* GABAergic interneurons as well as inhibit dopaminergic neurons directly [167].

A more recent experiment combining optogenetic modulation and slice recordings reported that the specific activation of vlPAG neurons induced glutamatergic excitatory post-synaptic currents in the majority of neurons (68%) in the VTA and inhibitory post-synaptic currents in a smaller proportion of neurons (50%), with 26% of the cells tested receiving both excitatory and inhibitory connections [168]. Unlike Omelchenko and Sesack [167], Waung and colleagues [168] found that only a minority of optogenetically activated cells in the VTA were TH+ (3/11 cells labeled with biocytin). The authors have also found that vlPAG - VTA neurons are activated in a mouse migraine model and inhibiting this pathway relieves the aversive state elicited during headache in mice [168]. Further work is now needed to fully evaluate the nature and influence of the periaqueductal grey nucleus over the VTA, considering that Ntamati and colleagues [165] also reported an excitatory projection to both dopaminergic and GABAergic cells in the VTA [165]. Similar studies are also required on the link between the periaqueductal grey nucleus and the SNc to test whether the periaqueductal GABAergic inputs can directly induce inhibitory responses in dopaminergic neurons following nociceptive stimuli or after a negative outcome described previously.

The literature on the link between the periaqueductal grey nucleus and the subthalamic nucleus can, to our knowledge, only be found in a single tract-tracing experiment providing evidence of a direct bilateral projection between these two structures [24]. The topography and role of this pathway should also be further explored according to the periaqueductal grey nucleus subdivisions. Similar to the parabrachial nucleus, the periaqueductal grey nucleus may also provide survival and defense signals to the subthalamic nucleus as part of the interrupt circuitry in case of an emergency, aspects of the avoidance/escape repertoire, which we suggest are not transmitted by the superior colliculus. The pathways linking the periaqueductal grey nucleus to the SNc/VTA and subthalamic nucleus are illustrated in Figs. (**3B** and **C**).

### 5.4 Periaqueductal Grey Nucleus Link with the Basal Ganglia - Summary

In summary, the link between the periaqueductal grey nucleus and the basal ganglia is also in crucial need of further investigation. There is strong evidence of functionally segregated channel-like loops, possibly sending bids to the basal ganglia to get access to common motor resources when survival is involved. The periaqueductal grey nucleus is also in a position to directly inhibit dopaminergic neurons *via* GABAergic modulation, at least for the VTA, possibly providing a solution to the need for the fast transmission of signals relating to negative/nociceptive events.

## CONCLUSION

So, evidence suggests that systems subserving approach and avoidance/escape are kept largely separate as they enter the striatum *via* the thalamus, at least in rats and mice, consistent with the non-overlapping behavioural demands of the eliciting contexts. Presumably, ecological pressures have resulted in the partitioning of these two sets of signals at the level of the superior colliculus, where each is involved in the formation of a partially closed loop. Further work is required to explore in more detail whether similar separated processing streams are present in higher species. In parallel with the separated signals feeding forward from the colliculus, the structure also appears to give rise to outputs that innervate other aspects of the basal ganglia, namely the SNc and subthalamic nucleus. These afferents arise, at least in the rat, from an area of the colliculus that appears to be involved in approach behavior. In terms of the afferents to dopaminergic neurons in the SNc, this accords with their role in reinforcement learning. It is also tempting to ascribe an approach function to the projections of the subthalamic nucleus. However, future studies are needed that manipulate this pathway optogenetically to convincingly assay its behavioural role. Unlike the segregated inputs to the striatum from the colliculus *via* the thalamus, the SNc and subthalamic nucleus seem to be pointed at which approach and avoidance/escape signals converge. Hence, dopaminergic neurons in the SNc receive nociceptive signals from the parabrachial nucleus and defense-related inputs from the periaqueductal grey nucleus, as does the subthalamic nucleus, and both dopaminergic neurons and the subthalamic nucleus receive appetitive signals from the colliculus. As with the convergence of the dorsal and ventral streams of cortical visual processing [169], it may make functional sense to unite the approach and avoidance/escape signals. Both are potential reasons to interrupt ongoing behaviour (*via* the subthalamic nucleus), and both are needed to feed a system that conveys information about events being better or worse than expected (the dopaminergic neurons in the SNc). Nonetheless, much work is still needed to answer some of the crucial outstanding questions. Why are dopaminergic neurons inhibited indirectly (*via* the RMTg) by negative stimuli? Related to that, what is the role of the direct glutamatergic parabrachial-VTA/SNc pathway that runs parallel to connections relaying in the RMTg/tail of the VTA? Indeed, the functional link between the basal ganglia and the parabrachial nucleus, or the periaqueductal grey nucleus and the basal ganglia, in the context of the response to potential threats, to our knowledge, has never been considered and studied. Whilst we clearly have some of the answers to how approach and avoidance/escape signals are processed by the basal ganglia, there is still much to learn.

## Figures and Tables

**Fig. (1):**
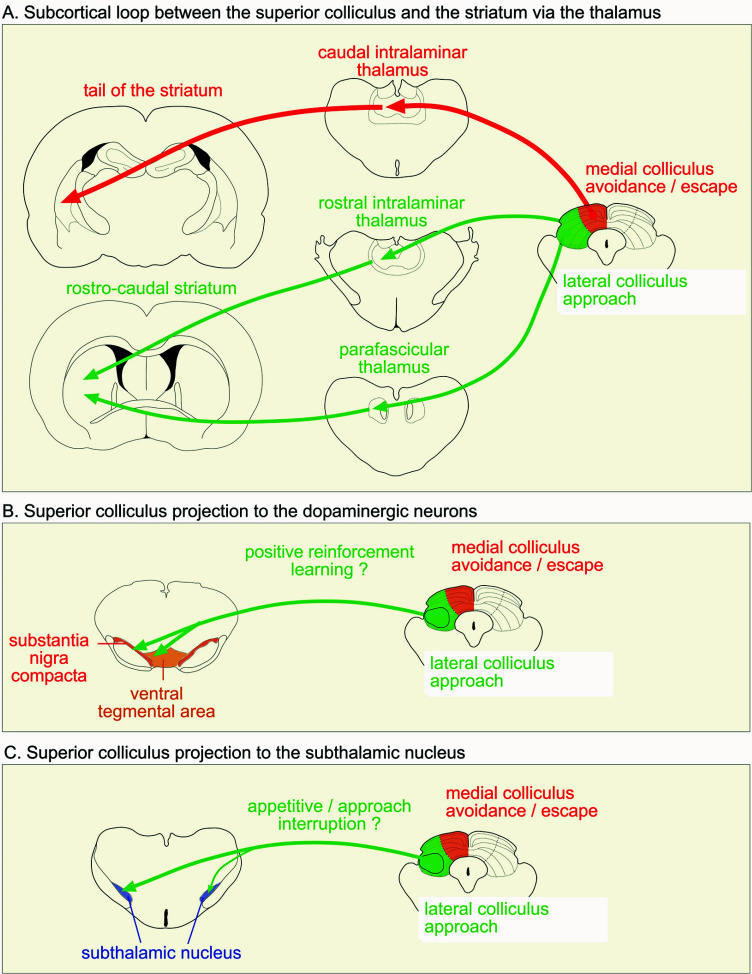
Schematic illustration of the projections from the superior colliculus to the striatum *via* the thalamus (**A**) (note that the densest projections are represented), to the dopaminergic neurons (**B**) and to the subthalamic nucleus (**C**).

**Fig. (2):**
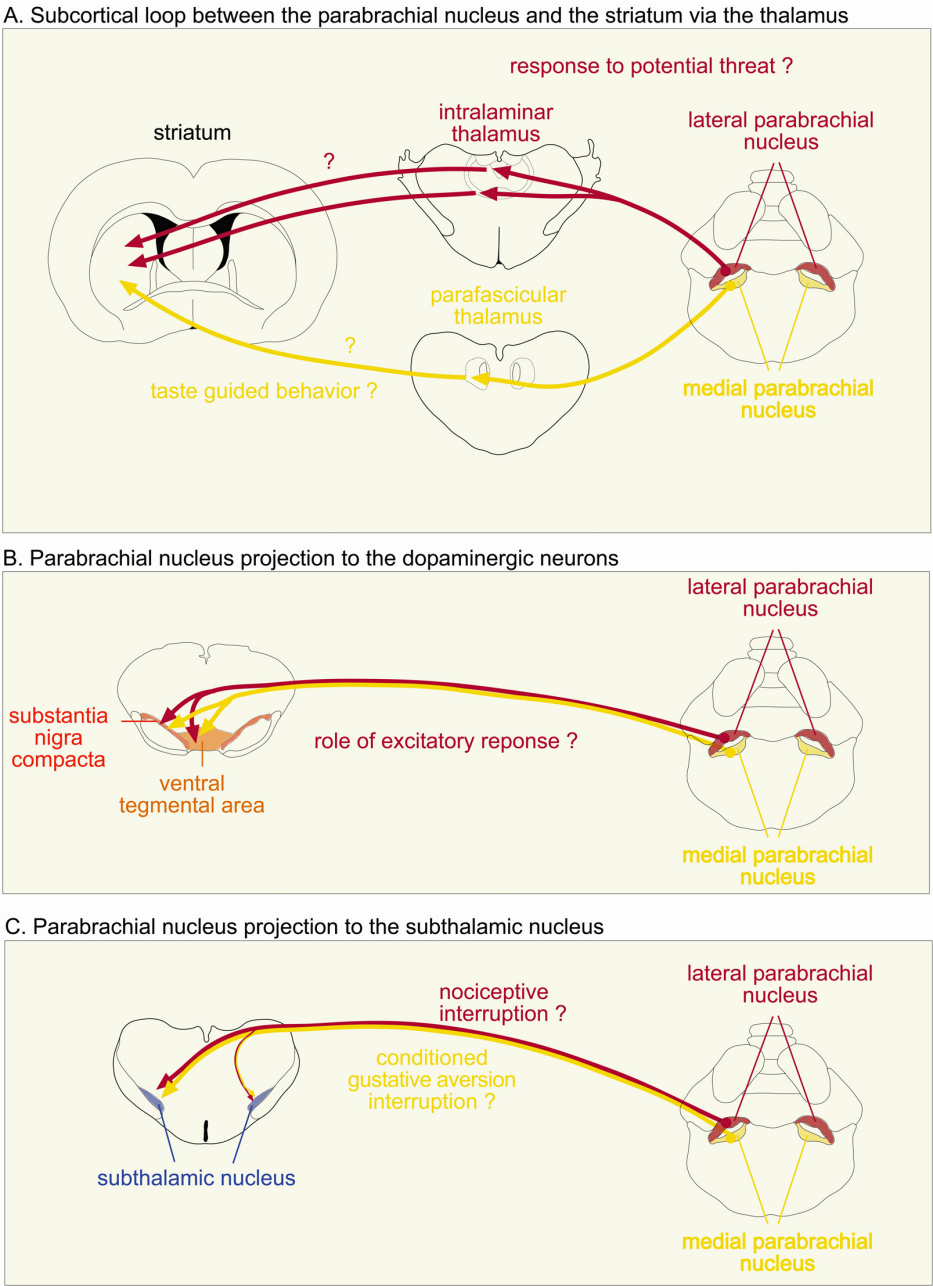
Schematic illustration of the projections from the parabrachial nucleus to the striatum *via* the thalamus (**A**) (note that the densest projections are represented), to the dopaminergic neurons (**B**) and to the subthalamic nucleus (**C**).

**Fig. (3):**
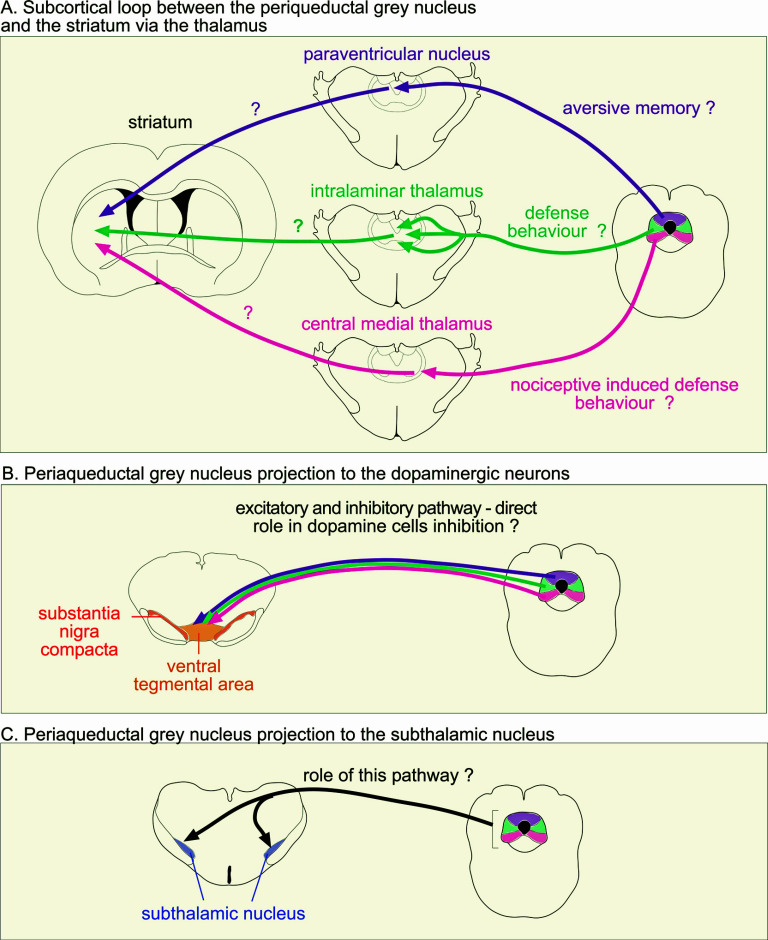
Schematic illustration of the projections from the periaqueductal grey nucleus to the striatum *via* the thalamus (**A**) (note that the densest projections are represented), to the dopaminergic neurons (**B**) and to the subthalamic nucleus (**C**).

## LIST OF ABBREVIATIONS

BDA: Biotinylated Dextran Amine
CGRP: Calcitonin Gene-related Peptide
dlPAG: Dorsolateral Periaqueductal Grey Nucleus
dmPAG: Dorsomedial Periaqueductal Grey Nucleus
lPAG: Lateral Periaqueductal Grey Nucleus
PHA-L: Phaseolus Vulgaris Leucoagglutinin
RMTg: Rostromedial Tegmental Nucleus
SNc: Substantia Nigra Pars Compacta
SNr: Substantia Nigra Pars Reticulate
TH: Tyrosine Hydroxylase
vlPAG: Ventrolateral Periaqueductal Grey Nucleus
VTA: Ventral Tegmental Area
